# Alteration of DNA Damage Response Causes Cleft Palate

**DOI:** 10.3389/fphys.2021.649492

**Published:** 2021-03-29

**Authors:** Hiroyuki Yamaguchi, Kohei Kitami, Xiao Wu, Li He, Jianbo Wang, Bin Wang, Yoshihiro Komatsu

**Affiliations:** ^1^Department of Pediatrics, The University of Texas Medical School at Houston, Houston, TX, United States; ^2^Department of Genetics, The University of Texas MD Anderson Cancer Center, Houston, TX, United States; ^3^Graduate Program in Genetics & Epigenetics, The University of Texas MD Anderson Cancer Center UTHealth Graduate School of Biomedical Sciences, Houston, TX, United States

**Keywords:** BRCA1, BRCA2, cleft palate, DNA damage response, poly (ADP-ribose) polymerase inhibitor

## Abstract

Cleft palate is one of the most common craniofacial birth defects, however, little is known about how changes in the DNA damage response (DDR) cause cleft palate. To determine the role of DDR during palatogenesis, the DDR process was altered using a pharmacological intervention approach. A compromised DDR caused by a poly (ADP-ribose) polymerase (PARP) enzyme inhibitor resulted in cleft palate in wild-type mouse embryos, with increased DNA damage and apoptosis. In addition, a mouse genetic approach was employed to disrupt breast cancer 1 (BRCA1) and breast cancer 2 (BRCA2), known as key players in DDR. An ectomesenchymal-specific deletion of *Brca1* or *Brca2* resulted in cleft palate due to attenuation of cell survival. This was supported by the phenotypes of the ectomesenchymal-specific *Brca1*/*Brca2* double-knockout mice. The cleft palate phenotype was rescued by superimposing p53 null alleles, demonstrating that the BRCA1/2–p53 DDR pathway is critical for palatogenesis. Our study highlights the importance of DDR in palatogenesis.

## Introduction

Cleft palate occurs in about one in every 500∼700 live births (Stanier and Moore, 2004; Dixon et al., 2011). Surgical repair is effective and currently the major treatment option. However, affected individuals still face serious functional and social challenges throughout their lives, with cleft palate imposing tremendous psychological and financial burdens on patients and their families. During the last decades, tremendous progress has been made toward understanding the genetic control(s) of this craniofacial abnormality (Murray, 2002; Bush and Jiang, 2012; Marazita, 2012). These studies clearly showed that genetic and cellular signaling pathways are tightly linked during palatogenesis, but the molecular details of cleft palate remain elusive. To overcome these difficulties, it is critical to uncover novel etiological mechanisms underlying cleft palate, enabling us to prevent and develop potential therapeutic strategies for this malformation.

In this regard, recent studies, including ours, reveal the importance of the DNA damage response (DDR) during craniofacial development (Sakai et al., 2016; Calo et al., 2018; Kitami et al., 2018). The DDR encompasses multiple DNA repair and signaling pathways, damage tolerance processes, and cell-cycle checkpoints, which safeguard genomic stability and integrity (McKinnon and Caldecott, 2007; Venkitaraman, 2014). Among craniofacial abnormalities, Treacher Collins syndrome is one of the many great examples that link a dysfunction in DDR to craniofacial defects (Sakai and Trainor, 2016; Sakai et al., 2016). Patients with heterozygous mutations in *TCOF1* present multiple craniofacial defects (Dixon et al., 2006; Jones et al., 2008); importantly, approximately 40–50% of *Tcof1*^+/–^ mice exhibit cleft palate and/or a high-arched palate (also known as “pseudo-cleft”) (Dixon et al., 2006; Conley et al., 2016), suggesting that an intact DDR is required for craniofacial development. To support this notion, we found that key components of the DDR, including tumor suppressor genes breast cancer 1 (BRCA1) and breast cancer 2 (BRCA2), are required for craniofacial bone development (Kitami et al., 2018). During early mouse embryogenesis, Treacle associates with the MRNM (MRE11, Rad51, NBS1, and MDC1) complex known as a DNA damage sensor and plays a critical role in DDR to limit oxidative stress induced neuroepithelial cell death (Sakai and Trainor, 2016; Sakai et al., 2016). Interestingly, *Brca1*-labeled DNA damage-induced foci are significantly reduced in *Tcof1*^+/–^ cells. Therefore, Treacle plays an important role in the DDR/repair through BRCA1 recruitment to and/or maintenance at sites of DNA damage, but Treacle may only interact with BRCA1 indirectly. Mutation of *Tcof1* results in severe craniofacial defects including cleft palate and hypoplasia of the facial bones, which represent several phenotypic similarities observed in neural crest-specific *Brca1* mutant mice (Kitami et al., 2018). Therefore, while definitive proof is still required, DDR regulated by both Treacle–BRCA1 axis may play a pivotal role in craniofacial development. These studies reinforce the idea that exploring this previously unappreciated DDR component in craniofacial abnormalities is critical for understanding how DDR functions in palatogenesis.

The aim of this study is to investigate the mechanistic connection(s) between a dysfunctional DDR, due to disruption of BRCA1/BRCA2 and cleft palate, using pharmacological and mouse genetic approaches to dissect the etiology of this craniofacial malformation.

## Results

### Treatment With a PARP Inhibitor Causes Cleft Palate in Mice

**FIGURE 1:**
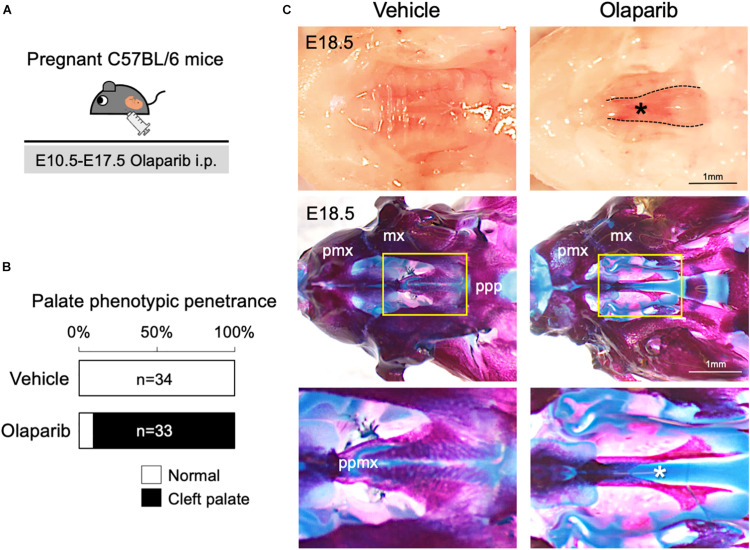
Treatment with PARP inhibitor Olaparib causes cleft palate in mice. **(A)** Schematic representation of the *in vivo* dosing of Olaparib (50 mg/kg) and tissue harvesting schedule. **(B)** Phenotypic penetrance of cleft palate at E16.5 following i.p. injection of Olaparib (50 mg/kg) into pregnant C57BL/6 mice. **(C)** Gross morphology of cleft palate (asterisk, upper panels) and skeletal analysis (middle panels) in embryos harvested from C57BL/6 pregnant females injected with Olaparib. The lower panel shows the high-magnification images of the area highlighted in the yellow boxes in the middle panel. mx, maxilla; pmx, premaxilla; ppmx, palatal process of maxilla; ppp, palatal process of palatine.

Poly (ADP-ribose) polymerase (PARP) inhibitors have garnered much enthusiasm for the treatment of breast cancer, but the inhibitors themselves may also induce DNA damage (Okazaki and Moss, 1999; Nathanson and Domchek, 2011). Therefore, we hypothesized that administration of a PARP inhibitor to mice may compromise DDR and induce craniofacial defects. To examine the influence of an altered DDR during mouse embryonic development, we used PARP inhibitor Olaparib, which is frequently used for the treatment of ovarian and breast cancer (Nathanson and Domchek, 2011; Chen et al., 2018). Olaparib (50 mg/kg) was administrated daily *via* intraperitoneal injection (i.p.) to pregnant C57BL/6 mice, from embryonic day (E) 10.5–E17.5; embryos were then harvested and analyzed at E18.5 (Figure 1A). While Olaparib treatment caused minor embryonic delayed growth (data not shown), the treated embryos developed cleft palate (*n* = 30/33) (Figures 1B,C). Skeletal analysis revealed that the development of the palatine bone was severely attenuated in Olaparib-treated embryos (Figure 1C), and histological analysis confirmed that cleft palate was morphologically obvious by E14.5 (Supplementary Figure 1). Because Olaparib-treated embryos also displayed shorter mandibles, we speculated that the cleft palate observed in these embryos could be attributed to abnormal mandible formation. To examine this possibility, we measured the length of the maxilla and mandible in both vehicle- and Olaparib-treated embryos. The ratio of the maxilla-mandible length was comparable until E15.5 (Supplementary Figure 2), indicating that cleft palate developed earlier than the mandibular defects observed in Olaparib-treated embryos. These results suggest that a functionally intact DDR is required for normal palatogenesis in mice.

### Olaparib Treatment Causes Increased DNA Damage in the Palate

**FIGURE 2:**
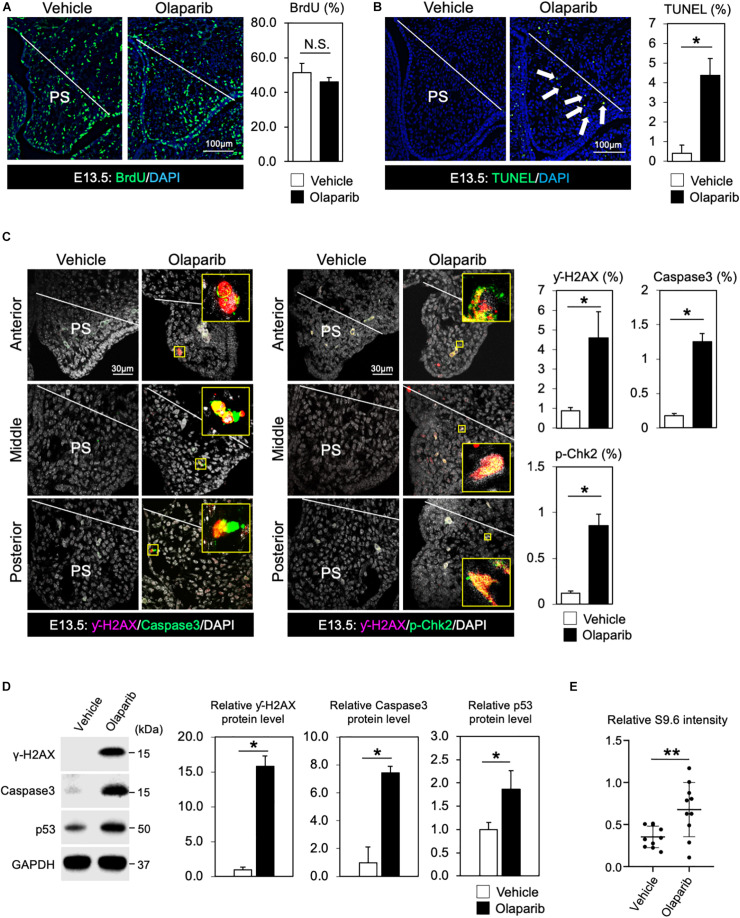
DDR is indispensable for regulating cell survival of palate tissues. **(A)** Coronal palate sections of vehicle- or Olaparib-treated mouse embryos were labeled with BrdU (green) to detect proliferative cells, which were quantified. PS, palatal shelf. **(B)** TUNEL assay (green) and corresponding quantification of palate sections from vehicle- or Olaparib-treated mouse embryos. Arrows show TUNEL-positive signals. PS, palatal shelf. **(C)** Immunostaining for γ-H2AX (magenta, left panels), Caspase3 (Green, left panels) and γ-H2AX (magenta, right panels), p-Chk2 (green, right panels), and corresponding quantification in palate sections from vehicle- or Olaparib-treated mouse embryos at E13.5. The yellow boxes show the high-magnification image of γ-H2AX/Caspase3 and γ-H2AX/p-Chk2-positive cells. PS, palatal shelf. **(D)** Protein levels of γ-H2AX, Caspase3 and p53 were examined by Western blotting and quantified. **(E)** S9.6 intensity was examined using palatal cell lysates from vehicle- or Olaparib-treated mouse embryos at E13.5. Data in panels **(A–D)** are represented as mean ± SD, *n* = 3 in each group. Data in panel **(E)** are represented as mean ± SD, *n* = 10 in each group. *p < 0.05; **p < 0.01; N.S., not significant.

In addition to its role in the basic excision repair in response to single stranded breaks, PARP also plays critical roles in other repair mechanisms and DNA replication, thus inhibition of PARP will lead to a compromised DDR. Therefore, Olaparib traps PARP on DNA and blocks DNA replication and transcription, leading to DNA breaks that prevent palatogenesis. To explore this hypothesis, we first examined cell proliferation activity and cell survival in Olaparib-treated mouse embryos. While cell proliferation activity was comparable between vehicle- and Olaparib-treated palate tissues (Figure 2A), a large number of apoptotic cells was detected in Olaparib-treated palate tissues (Figure 2B). This finding is consistent with other mouse models displaying cleft palate due to the massive amount of cell death in palatal mesenchymal cells (Liu et al., 2008, 2015; Goudy et al., 2010). To understand the etiology of cleft palate induced by Olaparib in wild-type embryos, we performed γ-H2AX/Caspase3 and γ-H2AX/phospho (p)-Chk2 marker analysis. Interestingly, over 80% of γ-H2AX positive cells were co-labeled with Caspase3 and/or p-Chk2 (Figure 2C). This suggests that treatment of Olaparib in wild-type embryos may cause DNA damage with our dosing condition. We confirmed that the protein levels of both γ-H2AX and Caspase3 were significantly increased, as well as p53 levels, in the palate tissues of Olaparib-treated embryos at E13.5 (Figure 2D). Because RNA-DNA hybrids influence genomic instability, the consequences of the presence of R-loops were examined with an anti-DNA-RNA hybrid (S9.6) antibody. We observed that the intensity of the S9.6 signals was increased in Olaparib-treated palate tissues compared with the controls (Figure 2E). These data demonstrate that DDR plays an important role in the cell survival of palatal tissues during palatogenesis.

### Disruption of *Brca1* and *Brca2* in Neural Crest Cells Leads to Cleft Palate in Mice

**FIGURE 3:**
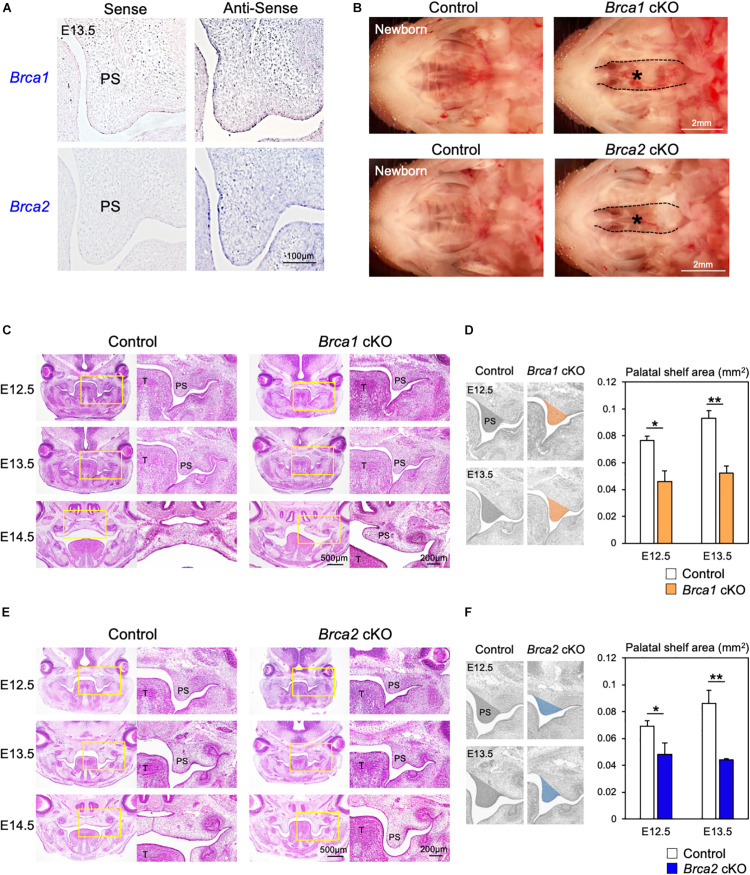
*Brca1* and *Brca2* in neural crest cells are critical for palatogenesis in mice. **(A)** *Brca1* and *Brca2* were expressed in mouse palate tissues. The corresponding sense probe was used as a negative control. PS, palatal shelf. **(B)** Neural crest-specific disruption of *Brca1* and *Brca2* induced cleft palate (asterisks). **(C)** H&E staining of sections from control and *Brca1* cKO embryos from E12.5 to E14.5. Asterisk shows cleft palate. PS, palatal shelf; T, tongue. **(D)** The quantification analysis of the palatal shelves area from control and *Brca1* cKO embryos at E12.5 and E13.5. PS, palatal shelf. **(E)** H&E staining of sections from control and *Brca2* cKO embryos from E12.5 to E14.5. Asterisk shows cleft palate. **(F)** The quantification analysis of the palatal shelves area from control and *Brca2* cKO embryos at E12.5 and E13.5. PS, palatal shelf. Data in panels **(D,F)** are represented as mean ± SD, *n* = 3 in each group. *p < 0.05; **p < 0.01.

The roles of the DDR have been studied extensively in cancer models (McKinnon and Caldecott, 2007; Riley et al., 2008; Venkitaraman, 2014), but little is known about their regulation and activity specifically during palatogenesis. This is partly because the deletion of DDR-associated genes frequently results in early embryonic lethality in mice (Gowen et al., 1996; Hakem et al., 1996; Suzuki et al., 1997; Xu et al., 1999). Therefore, it is not possible to explore how DDR functions during palate development. Since the DNA damage induced by Olaparib may cause cleft palate (Figures 1, 2), we asked which DDR pathways are predominantly involved in the etiology of this malformation. To understand the mechanisms of DDR in palatogenesis, we focused on key DDR elements BRCA1 and BRCA2, and designed RNA probes to examine the expression pattern of these genes in palate tissues in the mouse. Section *in situ* hybridization analysis showed that *Brca1* and *Brca2* were widely expressed in both palatal epithelial and mesenchymal cells (Figure 3A). Because epithelial-mesenchymal interactions are critical for palatogenesis (Bush and Jiang, 2012; He and Chen, 2012; Parada and Chai, 2012) and *Brca1* and *Brca2* are expressed in both palatal epithelial and mesenchymal cells (Figure 3A), we asked whether BRCA1 and BRCA2 function in these types of cells. First, we disrupted *Brca1* and *Brca2* in a neural crest-specific manner using a wingless-related MMTV integration site1 (*Wnt1*)-Cre driver line (Danielian et al., 1998; Xu et al., 1999). This approach allowed us to examine the function of BRCA1 and BRCA2 in neural crest-derived ectomesenchymal cells in the palate (Chai et al., 2000; Bronner and LeDouarin, 2012). We observed that mice lacking *Brca1* and/or *Brca2* in neural crest cells (“*Brca1* cKO” or “*Brca2* cKO” hereafter) displayed severe cleft palate with 100% phenotypic penetrance (Figure 3B). While *Brca1* cKO and *Brca2* cKO mutants were born at Mendelian ratios, they could not survive more than 24 h due to the cleft palate phenotype. Histological analysis confirmed that both *Brca1* cKO and *Brca2* cKO mutants showed cleft palate by E14.5 (Figures 3C–F). Next, we disrupted *Brca1* and *Brca2* using a Keratin14 (*K14*)-Cre driver line (Dassule et al., 2000), which allowed us to examine the function of BRCA1 and BRCA2 in palatal epithelial cells. In contrast to *Brca1* cKO and *Brca2* cKO mutants, mice with an epithelial cell-specific deletion of *Brca1* or *Brca2* did not show any overt craniofacial defects, including cleft palate (Supplementary Figure 3). These results indicate that BRCA1 and BRCA2 play a critical role during palatogenesis in murine neural crest-derived ectomesenchymal cells, but not in epithelial cells.

### Disruption of *Brca1* and *Brca2* Induces Palatal Cell Death

**FIGURE 4:**
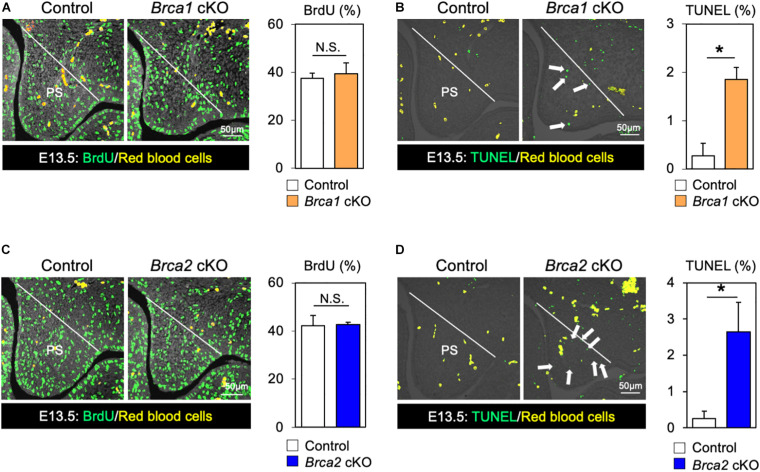
Disruption of *Brca1* and *Brca2* causes cell death in palate tissues. **(A)** Coronal palate sections of control and *Brca1* cKO embryos were labeled with BrdU (green) to detect proliferative cells, which were quantified. PS, palatal shelf. **(B)** TUNEL assay (green) and corresponding quantification in palate sections from control and *Brca1* cKO embryos. Arrows show TUNEL-positive signals. PS, palatal shelf. **(C)** Coronal palate sections of control and *Brca2* cKO embryos were labeled with BrdU (green) to detect proliferative cells, which were quantified. PS, palatal shelf. **(D)** TUNEL assay (green) corresponding quantification in palate sections from control and *Brca2* cKO embryos. Arrows show TUNEL-positive signals. PS, palatal shelf. Data are represented as mean ± SD, *n* = 3 in each group. *p < 0.05; N.S., not significant.

While *BRCA1* and *BRCA2* are known tumor suppressor genes, they may also function as regulators of cell proliferation and/or cell survival during mouse embryogenesis. Therefore, we asked whether the cleft palate observed in *Brca1* cKO and *Brca2* cKO mutants could be attributed to an absence of cell proliferation or excessive cell death. Whereas palatal mesenchymal cells showed similar proliferation activity in control and *Brca1* cKO mutant mice (Figure 4A), a large number of apoptotic cells were detected in the palate of *Brca1* cKO mutants (Figure 4B). Similar to *Brca1* cKO mutants, cell proliferation activity was comparable (Figure 4C), and cell survival was severely attenuated, in the palate of *Brca2* cKO compared to that of the controls (Figure 4D). Because cell death in pre-migratory neural crest cells frequently leads to craniofacial abnormalities (Noden and Trainor, 2005; Dixon et al., 2006), one might predict that increased cell death in pre-migratory neural crest cells may lead to cleft palate in *Brca1* cKO and *Brca2* cKO mutants. However, our recent findings show that BRCA1/2 is less likely to play a role in pre-migratory neural crest cells (Kitami et al., 2018). Altogether, these results suggest that the cleft palate phenotypes observed in *Brca1* cKO and *Brca2* cKO mutants are mainly due to excessive cell death, rather than a reduction in cell proliferation activity or defects in pre-migratory neural crests during craniofacial development.

### *Brca1* and *Brca2* Genes Function Together in Palatogenesis

**FIGURE 5:**
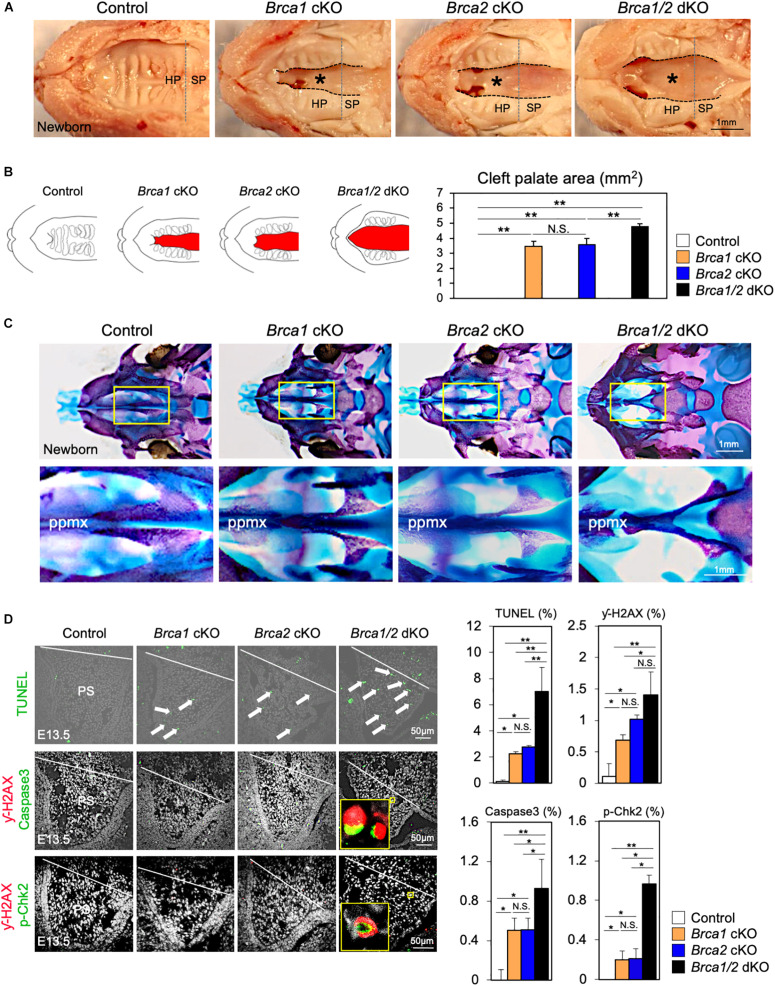
*Brca1* and *Brca2* function synergistically during palatogenesis in mice. **(A)** Gross morphology of cleft palate (asterisks) in *Brca1/2* mutants. HP, hard palate; SP, soft palate. **(B)** Schematic illustration of cleft palate (red) and the quantification of the palatal defective area among newborn control, *Brca1* cKO, *Brca2* cKO, and *Brca1/2* dKO mice. **(C)** Skeletal analysis of palatal bones in *Brca1/2* mutants. The lower panel shows the high-magnification images highlighted in the yellow boxes in the upper panel. ppmx, palatal process of maxilla. **(D)** TUNEL assay (green, upper panels) and corresponding quantification in palate sections from control, *Brca1* cKO, *Brca2* cKO and *Brca1/2* dKO mice. Arrows show TUNEL-positive signals. Immunostaining for γ-H2AX (magenta, middle panels) and Caspase3 (green, middle panels), γ-H2AX (magenta, lower panels) and p-Chk2 (green, lower panels), and corresponding quantification of palate sections from control, *Brca1* cKO, *Brca2* cKO, and *Brca1/2* dKO mice. The yellow box shows the high-magnification image of γ-H2AX/Caspase3- and γ-H2AX/p-Chk2-positive cells. PS, palatal shelf. Data in panels **(B,D)** are represented as mean ± SD, *n* = 3 in each group. *p < 0.05; **p < 0.01; N.S., not significant.

Breast cancer 1 plays a critical role in the response to DNA damage (Khanna and Jackson, 2001; Roy et al., 2011); additionally, BRCA2 is essential for maintaining genome integrity (McKinnon and Caldecott, 2007). Given the high similarity of both *Brca1* cKO and *Brca2* cKO cleft palate phenotypes (Figures 3, 4), it is important to examine whether *Brca1* and *Brca2* function redundantly during palatogenesis. To do so, we generated neural crest-specific *Brca1* and *Brca2* double- knockout mice (“*Brca1/2* dKO” hereafter). As in *Brca1* cKO and *Brca2* cKO mutants, deletion of both *Brca1* and *Brca2* in neural crest cells resulted in neonatal death (Supplementary Figure 4). Importantly, *Brca1/2* dKO mutants developed much more severe cleft palate phenotypes than the *Brca1* cKO and/or *Brca2* cKO mutants (Figure 5A). Measurement of the palatal defective area confirmed that the malformation seen in *Brca1/2* dKO mutants was very severe (Figure 5B), and skeletal analysis revealed that palatine bone formation was severely attenuated compared with *Brca1* cKO and/or *Brca2* cKO mutants (Figure 5C). To examine whether the disruption of both *Brca1* and *Brca2* in palatal tissues causes severe cell death, TUNEL analysis was performed. As expected, compared with *Brca1* cKO or *Brca2* cKO mutants, increased cell death was observed in *Brca1/2* dKO mutants (Figure 5D, upper panels). Consistent with the observation of excessive apoptosis in *Brca1/2* dKO mutants, the number of γ-H2AX-stained cells co-labeled with Caspase3 or p-Chk2 was significantly increased in *Brca1/2* dKO, even when compared with either the *Brca1* cKO or the *Brca2* cKO mutants (Figure 5D, middle and lower panels). These results suggest that while BRCA1 has multiple functions in the repair process of DNA damage and while BRCA2 plays a pivotal role in homologous recombination (HR), both *Brca1* and *Brca2* function synergistically during palatogenesis in mice.

### Deletion of *p53* Partially Rescues Cleft Palate by Decreasing DNA Damage and Preventing Cell Death in *Brca1* and *Brca2* Mutants

**FIGURE 6:**
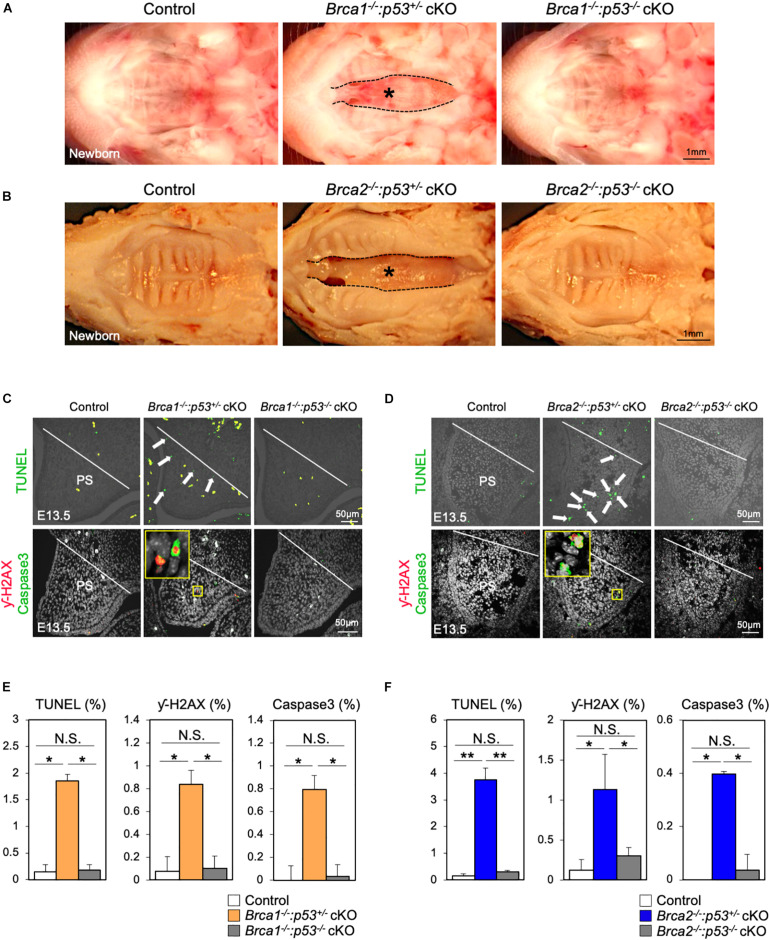
Deletion of *p53* partially rescues the cleft palate in *Brca1* and *Brca2* mutants. **(A)** Gross morphology of cleft palate in *Brca1* mutant mice (asterisk). Deletion of *p53* in *Brca1* cKO mice rescued the cleft palate phenotype. **(B)** Gross morphology of cleft palate in *Brca2* mutant mice (asterisk). Deletion of *p53* in *Brca2* cKO mice rescued the cleft palate phenotype. **(C)** TUNEL assay (green, upper panels) in palate sections from control, *Brca1*^–/–^:*p53*^+/–^ cKO, and *Brca1*^–/–^:*p53*^–/–^ cKO mice. Arrows show TUNEL-positive signals. Immunostaining for γ-H2AX (magenta, lower panels) and Caspase3 (green, lower panels) of palate sections. The yellow box shows the high-magnification image of γ-H2AX/Caspase3-positive cells. PS, palatal shelf. **(D)** TUNEL assay (green, upper panels) in palate sections from control, *Brca2*^–/–^:*p53*^+/–^ cKO, and *Brca2*^–/–^:*p53*^–/–^ cKO mice. Arrows show TUNEL-positive signals. Immunostaining for γ-H2AX (magenta, lower panels) and Caspase3 (green, lower panels) of palate sections. The yellow box shows the high-magnification image of γ-H2AX/Caspase3-positive cells. PS, palatal shelf. **(E)** Quantification of TUNEL-positive signals and γ-H2AX/Caspase3-positive cells in panel **(C)**. **(F)** Quantification of TUNEL-positive signals and γ-H2AX/Caspase3-positive cells in panel **(D)**. Data in panels **(E,F)** are represented as mean ± SD, *n* = 3 in each group. *p < 0.05; **p < 0.01; N.S., not significant.

It is known that DNA damage triggers p53 stabilization; if this damage cannot be repaired, it induces apoptosis (Riley et al., 2008). Therefore, we hypothesized that p53-mediated apoptosis may cause cleft palate in *Brca1* cKO and *Brca2* cKO mutants. Consistent with our hypothesis, the protein levels of p53 in palate tissues were much higher in *Brca1* cKO and *Brca2* cKO mutants than in the controls (Supplementary Figures 5A,B), suggesting that DNA damage-induced p53 stabilization may be responsible for the etiology of cleft palate in *Brca1* cKO and *Brca2* cKO mutants. To test this possibility *in vivo*, null alleles of *p53* were superimposed into *Brca1* cKO and *Brca2* cKO mutants in a neural crest-specific manner (Marino et al., 2000). Consistent with our prediction, the *p53* deletion rescued the cleft palate phenotype observed in *Brca1* cKO and *Brca2* cKO mutants (Figure 6A,B). Importantly, two copies of the *p53* deletion alleles were able to rescue the cleft palate more efficiently in *Brca1* cKO and *Brca2* cKO mutants than one copy (Supplementary Figures 5C,D). Phenotypic recovery was also confirmed by histological analysis (Supplementary Figure 6). We found that the *p53* deletion sufficiently suppressed the enhanced cell death seen in *Brca1* cKO and *Brca2* cKO palates (Figures 6C,D, upper panels, Figures 6E,F). This suppression of cell death in rescued mice was linked to a reduced number of γ-H2AX-stained cells co-labeled with Caspase3 (Figures 6C,D, lower panels, Figures 6E,F). Thus, reduction of *p53* levels in *Brca1* cKO and *Brca2* cKO mutants predominantly rescues the cleft palate by decreasing DNA damage-induced cell death. Altogether, these data demonstrate that BRCA1 and BRCA2 deficiency-induced cleft palate can be partially rescued by inactivating p53 through reduction of DNA damage-induced cell death.

## Discussion

The molecular etiology of cleft palate is very complex. Previous studies have suggested that altered transcriptional regulation, developmental signals, and/or epigenetic factors result in cleft palate. However, much still remains unclear about the regulation mechanisms of palate development. Recent studies, including ours, have shown the importance of the DDR during craniofacial development (Sakai et al., 2016; Calo et al., 2018; Kitami et al., 2018), but its role in normal palatogenesis is still not known. In this report, we found a novel connection between the etiology of cleft palate and the DDR, mainly *via* BRCA1/2-p53–dependent mechanisms. Our data indicate that maintaining genomic stability through the BRCA1 and BRCA2 DDR machinery may be key in preventing cleft palate.

### Temporospatial DDR Regulation Is Important for Palatogenesis

While a fine-tuned DDR machinery is essential for orchestrating morphological events, the action of the DDR during embryogenesis is highly temporal (Vinson and Hales, 2002; McKinnon, 2017). This is expected because, as for transcription and replication, DDR is a chromatin-associated process that is tightly regulated in a temporospatial manner. For example, although the *Brca1* and *Brca2* genes are expressed throughout the entire embryonic development process in mice, their expressions peak around E12.5–E13.5 in the craniofacial region (Lane et al., 1995; Rajan et al., 1997). Importantly, mid-gestation (i.e., E12.5–E13.5) is the stage when cellular metabolism switches from being anaerobic to being oxidative (Vinson and Hales, 2001, 2002), and this metabolic change may increase genotoxic stress in mouse embryos. Therefore, a considerable amount of DNA damage likely needs to be repaired in the craniofacial region around mid-gestation; if cells in this region fail to complete this task, severe craniofacial abnormalities may result. In support of these hypotheses, we recently found that the onset of craniofacial bone abnormalities in neural crest-specific *Brca1* and *Brca2* mutant mice indeed occurred around mid-gestation (Kitami et al., 2018), suggesting that temporospatial regulation of the DDR may be critically important for craniofacial development, including palatogenesis. Consistent with this prediction, the alterations in DDR, by either pharmacological interventions or genetic manipulation of DDR components analyzed in our study, caused cleft palate, further supporting our hypothesis that temporospatial DDR regulation is indispensable for palatogenesis.

To understand the mechanisms of DDR during palatogenesis, we focused on two important DDR elements: BRCA1 and BRCA2. Both genes are well-known DDR key players, critical for repairing DNA double-strand breaks to maintain genome integrity (McKinnon and Caldecott, 2007; Venkitaraman, 2014). While little is known about how DDR functions during palate development, recent studies have shown that while it is modest, there are BRCA1 and BRCA2 variants in non-syndromic cleft lip and palate patients (Kobayashi et al., 2013; Rodriguez et al., 2018). Another study also shows that the genetic variants in other DDR components including BRIP1 may contribute to the risk of non-syndromic cleft lip and palate (Mostowska et al., 2014). Therefore, it would be reasonable to speculate that BRCA1/2-dependent DDR may be linked to palatogenesis. Since we previously reported that both BRCA1 and BRCA2 are required for craniofacial bone formation (Kitami et al., 2018), these two DDR components provide us with a premise to examine whether dysregulation of DDR *via* the BRCA1 or the BRCA2 pathway leads to cleft palate. Importantly, conventional disruption of *Brca1* and *Brca2* in mice results in embryonic lethality, and this is mainly due to increased cell death and/or restricted proliferation in neuroepithelial cells (Gowen et al., 1996; Hakem et al., 1996; Suzuki et al., 1997; Xu et al., 2001). In addition, mice lacking *Brca1* and *Brca2* in neural stem cells display severe brain defects (Frappart et al., 2007; Pulvers and Huttner, 2009; Pao et al., 2014). These data indicate that BRCA1 and BRCA2 may play a pivotal role in neuroepithelial cells, which are critical for developing craniofacial structures such as palate tissues. Our findings support this notion that both BRCA1 and BRCA2 are required for the differentiation of neuroepithelial lineage cells, i.e., neural crest derivatives. Here we show that neural crest-specific disruption of *Brca1* and *Brca2* results in increased cell death in palatal mesenchymal cells, with the dual deletion of *Brca1/Brca2* leading to extensive apoptosis. Therefore, BRCA1 and BRCA2 in neural crest cells are most likely essential for the early onset of palatogenesis.

As in our neural crest-specific *Brca1* and *Brca2* mice, haploinsufficiency of the *Tcof1* mutation results in enhanced p53 production, and genetic suppression of *p53* in *Tcof1* mutant mice rescues the craniofacial bone defects (Jones et al., 2008). Of note, *Tcof1* haploinsufficiency causes neuroepithelial cell death, and loss of *Tcof1* decreases the accumulation of BRCA1 at DNA damage sites (Sakai et al., 2016), suggesting that TCOF1 and BRCA1 (and also possibly BRCA2) may be associated with the DDR during palatogenesis. It is worth noting that the *Tcof1* mutation results in cleft palate approximately 40–50% of the time, while the remaining *Tcof1*^+/–^ mice have a high arched palate (Dixon et al., 2006; Conley et al., 2016). It remains to be determined whether TCOF1, BRCA1, and BRCA2 genetically interact in palate development, and for this reason it is important to study how these specific DDR-related elements are involved in the etiology of cleft palate.

### BRCA1/2–Dependent DDR Is Critical for Palatogenesis

Whereas the importance of BRCA1/2-dependent DDR and repair has been adequately studied in tumorigenesis, how the BRCA1- and BRCA2-dependent DDR pathways function in palate development remains elusive. In addition to its role in regulating HR, BRCA1 acts in multiple aspects of DDR; on the other hand, BRCA2 facilitates HR. In this study, we found that neural crest-specific *Brca1* and *Brca2* mutants display an almost identical cleft palate phenotype. We initially hypothesized that a rapid neural crest cell proliferation may generate replication stress; this would render BRCA1 and BRCA2 essential to protect the replication fork’s integrity, with their deficiency leading to increased DNA damage during palate development. Indeed, it is known that BRCA1 and BRCA2 play a pivotal role in protecting the stability of a stalled replication fork to maintain genome integrity (Prakash et al., 2015). Surprisingly, the cell proliferation activity in the palatal mesenchyme in both *Brca1* and *Brca2* mutants was normal, demonstrating that DDR *via* BRCA1 or BRCA2 may be involved in alternative mechanisms other than regulating proliferation activity. In our study, increased cell death was predominantly involved in the etiology of cleft palate, and palatal defects were rescued by p53 reduction. Given the essential role of BRCA1 and BRCA2 in embryonic development and the identical cleft palate phenotypes in mice with neural crest-specific deletions of these genes, we conclude that BRCA1 and BRCA2 act redundantly during palatogenesis. Altogether, our data highlight the requirement of DDR, *via* BRCA1 and BRCA2, for normal palate development.

### PARP Inhibitor Therapy May Cause Fetal Cleft Palate

Breast cancer is the most common cancer diagnosed during pregnancy and affects about one in 3,000 pregnant women (Schedin, 2006; Letourneau et al., 2011). While curing breast cancer during pregnancy would be challenging, the use of PARP inhibitors has garnered much enthusiasm (Okazaki and Moss, 1999; Nathanson and Domchek, 2011), and several such agents have been approved by the FDA for the treatment of patients with ovarian and breast cancers harboring inherited BRCA1 and/or BRCA2 mutations. Since PARP is responsible for repairing single-strand breaks of damaged DNA, its inhibition leads to the formation of multiple double-strand DNA breaks (Lord et al., 2015; Chen et al., 2018). However, there is no indication whether pregnant women with breast cancer can be treated safely with these drugs, and little is known about the development of birth defects deriving from their use during pregnancy. In our study, we found that treatment with Olaparib results in severe cleft palate in wild-type mouse embryos. Out data suggest that Olaparib traps PARP on DNA, thus blocking DNA replication and transcription leading to DNA breaks, therefore it may lead to cleft palate. To the best of our knowledge, this is the first evidence of treatment with a PARP inhibitor causing cleft palate. Therefore, exploring the mechanisms of DDR may provide insights into the prevention of cleft palate when PARP inhibitor therapy is used in pregnant patients.

In summary, we demonstrated the critical function of DDR in palate development by pharmacological interventional and genetic approaches and determined that BRCA1/2–p53 dependent pathways are key in the regulation of normal palatogenesis in mice.

## Materials and Methods

### Animals

*Brca1*-floxed mice (Xu et al., 1999), *Brca2*-floxed mice (Jonkers et al., 2001), *p53*-floxed mice (Marino et al., 2000), *Wnt1-Cre* mice (Danielian et al., 1998), and *K14-Cre* mice (Dassule et al., 2000) were obtained from the NCI/NIH and The Jackson Laboratory. All mice were maintained in the animal facility of The University of Texas Medical School at Houston. The experimental protocol was reviewed and approved by the Animal Welfare Committee and the Institutional Animal Care and Use Committee of The University of Texas Medical School at Houston.

### Treatment of Animals With PARP Inhibitor (Olaparib)

Olaparib (AZD2281, Selleck Chemicals) was dissolved in 5.7% DMSO (Rottenberg et al., 2008; Szabova et al., 2014; Henneman et al., 2015). A dose of 50 mg of the Olaparib per kg of body weight per day was injected intraperitoneally into C57BL/6 pregnant mice (12–16 weeks old). Olaparib dosing conditions were established based on the frequency of embryonic lethality and phenotypic penetration of cleft palate (Supplementary Table 1).

### Skeletal Preparations and Histological Analysis

Staining of craniofacial tissues with Alizarin red and Alcian blue was carried out as previously described (Noda et al., 2016). The length and area ratio of cleft palate were measured with the ImageJ software. Immunofluorescent staining and TUNEL assays of paraffin sections were performed as previously described (Noda et al., 2016). Pregnant females were injected intraperitoneally with BrdU (Invitrogen, BrdU labeling reagent; 1 mL/100 g body weight). The primary antibodies used in immunofluorescence staining were as follows: BrdU (1:100, Abcam; ab6326), Caspase3 (1:200, Cell Signaling; 9664), and γ-H2AX (1:200, Millipore; 05-636), p-Chk2 (1:200, Cell Signaling; 2661). Stained slides were observed under a laser scanning confocal microscope (Olympus FluoView FV1000) using the FV10-ASW Viewer software (version 4.2).

### Section *in situ* Hybridization

PCR fragments containing regions of mouse *Brca1* (nucleotides 2,302–3,028 bp) and mouse *Brca2* (nucleotides 162–653 bp) cDNA were cloned into the pCR^®^II vector. Digoxygenin-labeled sense- and/or antisense-*Brca1* and -*Brca2* probes were generated by *in vitro* transcription (Sigma-Aldrichı). Section *in situ* hybridization was performed using standard procedures (Komatsu et al., 2014).

### Western Blot Analysis

Palatal tissues were homogenized with protein lysis buffers. After centrifugation, the supernatants were separated by SDS/PAGE, blotted onto a PVDF membrane, and analyzed with specific antibodies. The antibodies used were as follows: GAPDH (1:5,000, Cell Signaling; 2118), p53 (1:2,000, Santa Cruz; sc-6243), Caspase3 (1:500, Cell Signaling; 9664), and γ-H2AX (1:500, Millipore; 05-636). The Clarity Max ECL Substrate (Bio-Rad) was used for chemiluminescent detection, and signals were quantified with the Image Lab Version 6.0 software (Bio-Rad).

### R-Loop Assay

DNA was extracted from vehicle- or Olaparib-treated palatal tissues at E13.5 (*n* = 10). DNA concentration was measured with a Nano-drop spectrophotometer, and 500 ng of DNA were applied onto a membrane using a dot blot apparatus. After cross-linking, the membrane was subjected to R-loop detection with the S9.6 antibody. S9.6 signals were normalized to the total DNA signals and quantified using the ImageJ software.

### Statistical Analysis

The Student’s *t* test and one-way ANOVA with *post hoc* Tukey HSD test were used for statistical analysis. A *p* value of less than 0.05 was considered statistically significant.

## Data Availability Statement

The raw data supporting the conclusions of this article will be made available by the authors, without undue reservation.

## Ethics Statement

The animal study was reviewed and approved by The Animal Welfare Committee and the Institutional Animal Care and Use Committee of The University of Texas Medical School at Houston.

## Author Contributions

YK designed the study. HY, KK, XW, LH, and JW performed the experiments. HY, KK, XW, BW, and YK analyzed the data. BW and YK wrote the manuscript. All authors contributed to the article and approved the submitted version.

## Conflict of Interest

The authors declare that the research was conducted in the absence of any commercial or financial relationships that could be construed as a potential conflict of interest.

## Supplementary Material

Supplementary Figure 1 Treatment with PARP inhibitor Olaparib causes cleft palate. **(A)** Gross morphology of cleft palate (asterisk) in embryos harvested from C57BL/6 pregnant mice injected with Olaparib (50 mg/kg). **(B)** H&E staining of sections from vehicle- or Olaparib-treated mouse embryos at E12.5, E13.5, E14.5, and E16.5. Asterisk shows cleft palate. **(C)** The quantification analysis of the palatal shelves area from vehicle- or Olaparib-treated mouse embryos at E12.5 and E13.5. Data in panel **(C)** are represented as mean ± SD, *n* = 3 in each group. ^∗∗^*p* < 0.01.

Supplementary Figure 2 The ratio of maxilla-mandible length of Olaparib-treated mouse embryos. **(A)** The landmarks of mandibular length (L) and maxillary length (U). Schematic illustration of a lateral superimposition of Olaparib-treated (red)/Vehicle-treated (blue) embryo at E18.5. **(B)** The analysis of maxilla (U-Vehicle: blue line, U-Olaparib: blue broken line) and mandible length (L-Vehicle: red line, L-Olaparib: red broken line), and maxilla-mandible ratio (white: L/U-Vehicle, black: L/U-Olaparib). Data are represented as mean ± SD, *n* = 6 in each group. ^∗^*p* < 0.05; N.S., not significant.

Supplementary Figure 3 Absence of cleft palate with epithelial cell-specific deletion of *Brca1* and *Brca2* in mice. **(A)** H&E staining of coronal section of control and *Brca1:K14-Cre* mice. **(B)** H&E staining of coronal section of control and *Brca2:K14-Cre* mice.

Supplementary Figure 4 Neonatal death in neural crest-specific *Brca1*, *Brca2*, and *Brca1/2* double knockout mice. Whole-mount view of control, *Brca1:Wnt1-Cre* (*Brca1* cKO), *Brca2:Wnt1-Cre* (*Brca2* cKO), and *Brca1:Brca2:Wnt1-Cre* (*Brca1/2* dKO) newborn mice.

Supplementary Figure 5 The BRCA1–p53 and BRCA2–p53 pathways play a pivotal role in palatogenesis. **(A,B)** Western blotting analysis of palatal tissue from E13.5 embryos. Each sample is from an individual embryo. The chart on the right shows the quantification of the relative p53 protein levels. **(C,D)** Quantification of the phenotypic penetration for each genotype. Data in panels **(A,B)** are represented as mean ± SD, *n* = 3 in each group. ^∗^*p* < 0.05.

Supplementary Figure 6 Deletion of p53 rescues cleft palate in *Brca1* and *Brca2* mutant mice. **(A)** H&E staining of coronal section of control, *Brca1*^–/–^:*p53*^+/–^ cKO, and *Brca1*^–/–^:*p53*^–/–^ cKO mice. Asterisk shows cleft palate. **(B)** H&E staining of coronal section of control, *Brca2*^–/–^:*p53*^+/–^ cKO, and *Brca2*^–/–^:*p53*^–/–^ cKO mice. Asterisk shows cleft palate.

Supplementary Table 1 Summary of cleft palate phenotypes following treatment with Olaparib at different timepoints. **(A)** 90.5% of Olaparib-treated embryos (50 mg/kg, E8.5–E15.5, daily) were dead at E16.5. **(B)** 90.9% of Olaparib-treated embryos (50 mg/kg, E10.5–E15.5, daily) were alive and showed cleft palate at E16.5. **(C,D)** All of the Olaparib-treated embryos (50 mg/kg, E11.5 or E12.5–E15.5, daily) were alive and showed normal palate development at E16.5. **(E,F)** All of the vehicle-treated embryos (5.7%DMSO, E8.5–E15.5 or E10.5–E15.5, daily) were alive and showed normal palate development at E16.5.

## References

[B1] Bronner M. E. LeDouarin N. M. (2012). Development and evolution of the neural crest: an overview. *Dev. Biol.* 366 2–9. 10.1016/j.ydbio.2011.12.042 22230617 PMC3351559

[B2] Bush J. O. Jiang R. (2012). Palatogenesis: morphogenetic and molecular mechanisms of secondary palate development. *Development* 139 231–243. 10.1242/dev.067082 22186724 PMC3243091

[B3] Calo E. Gu B. Bowen M. E. Aryan F. Zalc A. Liang J. (2018). Tissue-selective effects of nucleolar stress and rDNA damage in developmental disorders. *Nature* 554 112–117. 10.1038/nature25449 29364875 PMC5927778

[B4] Chai Y. Jiang X. Ito Y. Bringas P. Jr. Han J. Rowitch D. H. (2000). Fate of the mammalian cranial neural crest during tooth and mandibular morphogenesis. *Development* 127 1671–1679. 10725243 10.1242/dev.127.8.1671

[B5] Chen C. C. Feng W. Lim P. X. Kass E. M. Jasin M. (2018). Homology-Directed Repair and the Role of BRCA1, BRCA2, and Related Proteins in Genome Integrity and Cancer. *Annu. Rev. Cancer Biol.* 2 313–336. 10.1146/annurev-cancerbio-030617-050502 30345412 PMC6193498

[B6] Conley Z. R. Hague M. Kurosaka H. Dixon J. Dixon M. J. Trainor P. A. (2016). A quantitative method for defining high-arched palate using the Tcof1(+/-) mutant mouse as a model. *Dev. Biol.* 415 296–305. 10.1016/j.ydbio.2015.12.020 26772999 PMC4914414

[B7] Danielian P. S. Muccino D. Rowitch D. H. Michael S. K. McMahon A. P. (1998). Modification of gene activity in mouse embryos in utero by a tamoxifen-inducible form of Cre recombinase. *Curr. Biol. CB* 8 1323–1326. 9843687 10.1016/s0960-9822(07)00562-3

[B8] Dassule H. R. Lewis P. Bei M. Maas R. McMahon A. P. (2000). Sonic hedgehog regulates growth and morphogenesis of the tooth. *Dev.* 127 4775–4785. 10.1242/dev.127.22.4775 11044393

[B9] Dixon J. Jones N. C. Sandell L. L. Jayasinghe S. M. Crane J. Rey J. P. (2006). Tcof1/Treacle is required for neural crest cell formation and proliferation deficiencies that cause craniofacial abnormalities. *Proc. Natl. Acad. Sci. U S A.* 103 13403–13408. 10.1073/pnas.0603730103 16938878 PMC1557391

[B10] Dixon M. J. Marazita M. L. Beaty T. H. Murray J. C. (2011). Cleft lip and palate: understanding genetic and environmental influences. *Nat. Rev. Genet.* 12 167–178. 10.1038/nrg2933 21331089 PMC3086810

[B11] Frappart P. O. Lee Y. Lamont J. McKinnon P. J. (2007). BRCA2 is required for neurogenesis and suppression of medulloblastoma. *EMBO J.* 26 2732–2742. 10.1038/sj.emboj.7601703 17476307 PMC1888666

[B12] Goudy S. Law A. Sanchez G. Baldwin H. S. Brown C. (2010). Tbx1 is necessary for palatal elongation and elevation. *Mech. Dev.* 127 292–300. 10.1016/j.mod.2010.03.001 20214979 PMC2871954

[B13] Gowen L. C. Johnson B. L. Latour A. M. Sulik K. K. Koller B. H. (1996). Brca1 deficiency results in early embryonic lethality characterized by neuroepithelial abnormalities. *Nat. Genet.* 12 191–194. 10.1038/ng0296-191 8563759

[B14] Hakem R. de la Pompa J. L. Sirard C. Mo R. Woo M. Hakem A. (1996). The tumor suppressor gene Brca1 is required for embryonic cellular proliferation in the mouse. *Cell* 85 1009–1023. 8674108 10.1016/s0092-8674(00)81302-1

[B15] He F. Chen Y. (2012). Wnt signaling in lip and palate development. *Front. Oral Biol.* 16:81–90. 10.1159/000337619 22759672

[B16] Henneman L. van Miltenburg M. H. Michalak E. M. Braumuller T. M. Jaspers J. E. Drenth A. P. (2015). Selective resistance to the PARP inhibitor olaparib in a mouse model for BRCA1-deficient metaplastic breast cancer. *Proc. Natl. Acad. Sci. U S A.* 112 8409–8414. 10.1073/pnas.1500223112 26100884 PMC4500240

[B17] Jones N. C. Lynn M. L. Gaudenz K. Sakai D. Aoto K. Rey J. P. (2008). Prevention of the neurocristopathy Treacher Collins syndrome through inhibition of p53 function. *Nat. Med.* 14 125–133. 10.1038/nm1725 18246078 PMC3093709

[B18] Jonkers J. Meuwissen R. van der Gulden H. Peterse H. van der Valk M. Berns A. (2001). Synergistic tumor suppressor activity of BRCA2 and p53 in a conditional mouse model for breast cancer. *Nat. Genet.* 29 418–425. 10.1038/ng747 11694875

[B19] Khanna K. K. Jackson S. P. (2001). DNA double-strand breaks: signaling, repair and the cancer connection. *Nat. Genet.* 27 247–254. 10.1038/85798 11242102

[B20] Kitami K. Kitami M. Kaku M. Wang B. Komatsu Y. (2018). BRCA1 and BRCA2 tumor suppressors in neural crest cells are essential for craniofacial bone development. *PLoS Genet.* 14:e1007340. 10.1371/journal.pgen.1007340 29718910 PMC5951594

[B21] Kobayashi G. S. Alvizi L. Sunaga D. Y. Francis-West P. Kuta A. Almada B. V. (2013). Susceptibility to DNA damage as a molecular mechanism for non-syndromic cleft lip and palate. *PLoS One* 8:e65677. 10.1371/journal.pone.0065677 23776525 PMC3680497

[B22] Komatsu Y. Kishigami S. Mishina Y. (2014). In situ hybridization methods for mouse whole mounts and tissue sections with and without additional beta-galactosidase staining. *Methods Mol. Biol.* 1092 1–15. 10.1007/978-1-60327-292-6_1 24318810 PMC4118283

[B23] Lane T. F. Deng C. Elson A. Lyu M. S. Kozak C. A. Leder P. (1995). Expression of Brca1 is associated with terminal differentiation of ectodermally and mesodermally derived tissues in mice. *Genes Dev.* 9 2712–2722. 7590247 10.1101/gad.9.21.2712

[B24] Letourneau J. M. Melisko M. E. Cedars M. I. Rosen M. P. (2011). A changing perspective: improving access to fertility preservation. *Nat. Rev. Clin. Oncol.* 8 56–60. 10.1038/nrclinonc.2010.133 20736926 PMC3226819

[B25] Liu W. Lan Y. Pauws E. Meester-Smoor M. A. Stanier P. Zwarthoff E. C. (2008). The Mn1 transcription factor acts upstream of Tbx22 and preferentially regulates posterior palate growth in mice. *Development* 135 3959–3968. 10.1242/dev.025304 18948418 PMC2586179

[B26] Liu Y. Wang M. Zhao W. Yuan X. Yang X. Li Y. (2015). Gpr177-mediated Wnt Signaling Is Required for Secondary Palate Development. *J. Dent. Res.* 94 961–967. 10.1177/0022034515583532 25922332

[B27] Lord C. J. Tutt A. N. Ashworth A. (2015). Synthetic lethality and cancer therapy: lessons learned from the development of PARP inhibitors. *Annu. Rev. Med.* 66 455–470. 10.1146/annurev-med-050913-022545 25341009

[B28] Marazita M. L. (2012). The evolution of human genetic studies of cleft lip and cleft palate. *Annu. Rev. Genomics Hum. Genet.* 13 263–283. 10.1146/annurev-genom-090711-163729 22703175 PMC3760163

[B29] Marino S. Vooijs M. van Der Gulden H. Jonkers J. Berns A. (2000). Induction of medulloblastomas in p53-null mutant mice by somatic inactivation of Rb in the external granular layer cells of the cerebellum. *Genes Dev.* 14 994–1004. 10783170 PMC316543

[B30] McKinnon P. J. (2017). Genome integrity and disease prevention in the nervous system. *Genes Dev.* 31 1180–1194. 10.1101/gad.301325.117 28765160 PMC5558921

[B31] McKinnon P. J. Caldecott K. W. (2007). DNA strand break repair and human genetic disease. *Annu. Rev. Genomics Hum. Genet.* 8 37–55. 10.1146/annurev.genom.7.080505.115648 17887919

[B32] Mostowska A. Hozyasz K. K. Wójcicki P. Galas-Filipowicz D. Lasota A. Dunin-Wilczyńska I. (2014). Genetic variants in BRIP1 (BACH1) contribute to risk of nonsyndromic cleft lip with or without cleft palate. *Birth Defects Res. Clin. Mol. Teratol.* 100 670–678. 10.1002/bdra.23275 25045080

[B33] Murray J. C. (2002). Gene/environment causes of cleft lip and/or palate. *Clin. Genet.* 61 248–256. 10.1034/j.1399-0004.2002.610402.x 12030886

[B34] Nathanson K. L. Domchek S. M. (2011). Therapeutic approaches for women predisposed to breast cancer. *Annu. Rev. Med.* 62 295–306. 10.1146/annurev-med-010910-110221 21034216

[B35] Noda K. Kitami M. Kitami K. Kaku M. Komatsu Y. (2016). Canonical and noncanonical intraflagellar transport regulates craniofacial skeletal development. *Proc. Natl. Acad. Sci. U S A.* 113 E2589–E2597. 10.1073/pnas.1519458113 27118846 PMC4868419

[B36] Noden D. M. Trainor P. A. (2005). Relations and interactions between cranial mesoderm and neural crest populations. *J. Anat.* 207 575–601. 10.1111/j.1469-7580.2005.00473.x 16313393 PMC1571569

[B37] Okazaki I. J. Moss J. (1999). Characterization of glycosylphosphatidylinositiol-anchored, secreted, and intracellular vertebrate mono-ADP-ribosyltransferases. *Annu. Rev. Nutr.* 19 485–509. 10.1146/annurev.nutr.19.1.485 10448534

[B38] Pao G. M. Zhu Q. Perez-Garcia C. G. Chou S. J. Suh H. Gage F. H. (2014). Role of BRCA1 in brain development. *Proc. Natl. Acad. Sci. U S A.* 111 E1240–E1248. 10.1073/pnas.1400783111 24639535 PMC3977248

[B39] Parada C. Chai Y. (2012). Roles of BMP signaling pathway in lip and palate development. *Front. Oral Biol.* 16:60–70. 10.1159/000337617 22759670 PMC3661199

[B40] Prakash R. Zhang Y. Feng W. Jasin M. (2015). Homologous recombination and human health: the roles of BRCA1, BRCA2, and associated proteins. *Cold Spring Harb. Perspect. Biol.* 7:a016600. 10.1101/cshperspect.a016600 25833843 PMC4382744

[B41] Pulvers J. N. Huttner W. B. (2009). Brca1 is required for embryonic development of the mouse cerebral cortex to normal size by preventing apoptosis of early neural progenitors. *Development* 136 1859–1868. 10.1242/dev.033498 19403657

[B42] Rajan J. V. Marquis S. T. Gardner H. P. Chodosh L. A. (1997). Developmental expression of Brca2 colocalizes with Brca1 and is associated with proliferation and differentiation in multiple tissues. *Dev. Biol.* 184 385–401. 10.1006/dbio.1997.8526 9133444

[B43] Riley T. Sontag E. Chen P. Levine A. (2008). Transcriptional control of human p53-regulated genes. *Nat. Rev. Mol. Cell Biol.* 9 402–412. 10.1038/nrm2395 18431400

[B44] Rodriguez N. Maili L. Chiquet B. T. Blanton S. H. Hecht J. T. Letra A. (2018). BRCA1 and BRCA2 gene variants and nonsyndromic cleft lip/palate. *Birth Defects Res.* 110 1043–1048. 10.1002/bdr2.1346 29921024 PMC6105370

[B45] Rottenberg S. Jaspers J. E. Kersbergen A. van der Burg E. Nygren A. O. Zander S. A. (2008). High sensitivity of BRCA1-deficient mammary tumors to the PARP inhibitor AZD2281 alone and in combination with platinum drugs. *Proc. Natl. Acad. Sci. U S A.* 105 17079–17084. 10.1073/pnas.0806092105 18971340 PMC2579381

[B46] Roy R. Chun J. Powell S. N. (2011). BRCA1 and BRCA2: different roles in a common pathway of genome protection. *Nat. Rev. Cancer* 12 68–78. 10.1038/nrc3181 22193408 PMC4972490

[B47] Sakai D. Trainor P. A. (2016). Face off against ROS: Tcof1/Treacle safeguards neuroepithelial cells and progenitor neural crest cells from oxidative stress during craniofacial development. *Dev. Growth Differentiat.* 58 577–585. 10.1111/dgd.12305 27481486 PMC5026570

[B48] Sakai D. Dixon J. Achilleos A. Dixon M. Trainor P. A. (2016). Prevention of Treacher Collins syndrome craniofacial anomalies in mouse models via maternal antioxidant supplementation. *Nat. Communicat.* 7:10328. 10.1038/ncomms10328 26792133 PMC4735750

[B49] Schedin P. (2006). Pregnancy-associated breast cancer and metastasis. *Nat. Rev. Cancer* 6 281–291. 10.1038/nrc1839 16557280

[B50] Stanier P. Moore G. E. (2004). Genetics of cleft lip and palate: syndromic genes contribute to the incidence of non-syndromic clefts. *Hum. Mol. Genet.* 13 R73–R81. 10.1093/hmg/ddh052 14722155

[B51] Suzuki A. de la Pompa J. L. Hakem R. Elia A. Yoshida R. Mo R. (1997). Brca2 is required for embryonic cellular proliferation in the mouse. *Genes Dev.* 11 1242–1252. 9171369 10.1101/gad.11.10.1242

[B52] Szabova L. Bupp S. Kamal M. Householder D. B. Hernandez L. Schlomer J. J. (2014). Pathway-specific engineered mouse allograft models functionally recapitulate human serous epithelial ovarian cancer. *PLoS One* 9:e95649. 10.1371/journal.pone.0095649 24748377 PMC3991711

[B53] Venkitaraman A. R. (2014). Cancer suppression by the chromosome custodians, BRCA1 and BRCA2. *Science* 343 1470–1475. 10.1126/science.1252230 24675954

[B54] Vinson R. K. Hales B. F. (2001). Nucleotide excision repair gene expression in the rat conceptus during organogenesis. *Mutat. Res.* 486 113–123. 10.1016/s0921-8777(01)00087-8 11425516

[B55] Vinson R. K. Hales B. F. (2002). DNA repair during organogenesis. *Mutat. Res.* 509 79–91. 12427532 10.1016/s0027-5107(02)00223-3

[B56] Xu X. Qiao W. Linke S. P. Cao L. Li W. M. Furth P. A. (2001). Genetic interactions between tumor suppressors Brca1 and p53 in apoptosis, cell cycle and tumorigenesis. *Nat. Genet.* 28 266–271. 10.1038/90108 11431698

[B57] Xu X. Wagner K. U. Larson D. Weaver Z. Li C. Ried T. (1999). Conditional mutation of Brca1 in mammary epithelial cells results in blunted ductal morphogenesis and tumour formation. *Nat. Genet.* 22 37–43. 10.1038/8743 10319859

