# Supplementary material for: Alteration of DNA Damage Response Causes Cleft Palate
Source: Front Physiol. 2021 Mar 29;12:649492. doi: 10.3389/fphys.2021.649492 (PMC8039291; doi:10.3389/fphys.2021.649492)
Supplement: Supplementary Figure 1 — Treatment with PARP inhibitor Olaparib causes cleft palate. (A) Gross morphology of cleft palate (asterisk) in embryos harvested from C57BL/6 pregnant mice injected with Olaparib (50 mg/kg). (B) H&E staining of sections from vehicle- or Olaparib-treated mouse embryos at E12.5, E13.5, E14.5, and E16.5. Asterisk shows cleft palate. (C) The quantification analysis of the palatal shelves area from vehicle- or Olaparib-treated mouse embryos at E12.5 and E13.5. Data in panel (C) are represented as mean ± SD, n = 3 in each group. ∗∗p < 0.01. [file Data_Sheet_1.pdf]

Supplementary Material

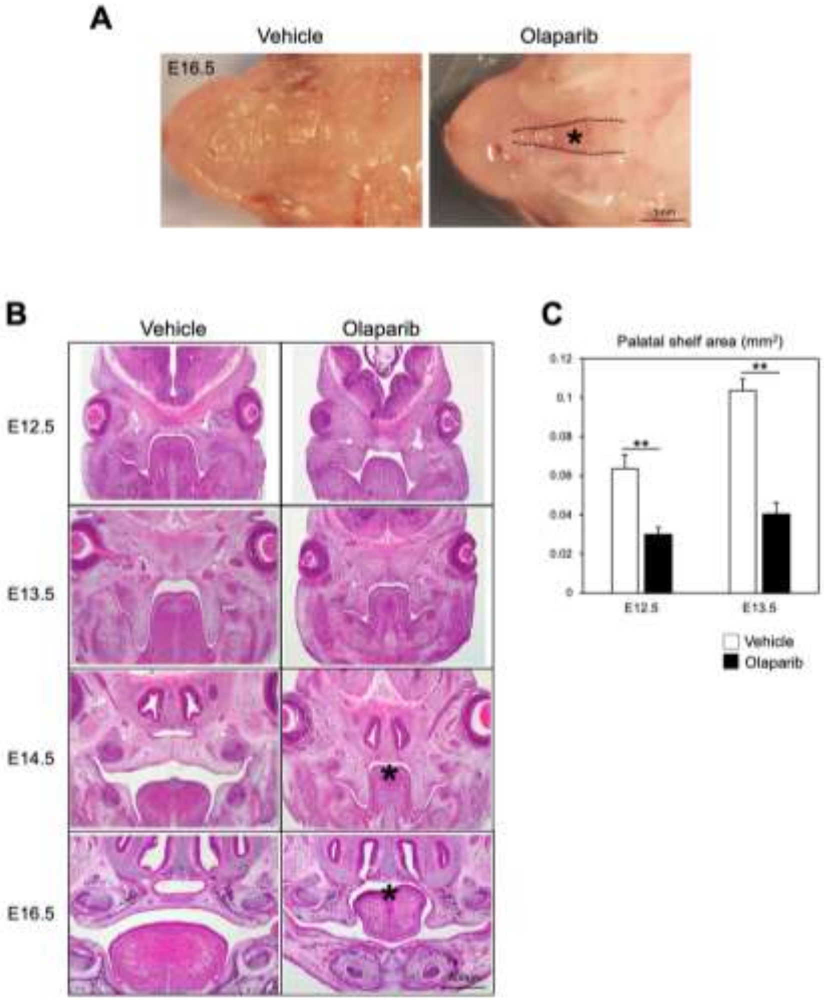

Supplementary Fig. 1. Treatment with PARP inhibitor Olaparib causes cleft palate.

(A) Gross morphology of cleft palate (asterisk) in embryos harvested from C57BL/6 pregnant mice injected with Olaparib (50mg/kg). **(B)** H&E staining of sections from vehicle- or Olaparib-treated mouse embryos at E12.5, E13.5, E14.5 and E16.5. Asterisk shows cleft palate. **(C)** The quantification analysis of the palatal shelves area from vehicle- or Olaparib-treated mouse embryos at E12.5 and E13.5. Data in (C) are represented as mean±SD, n=3 in each group. **p<0.01.

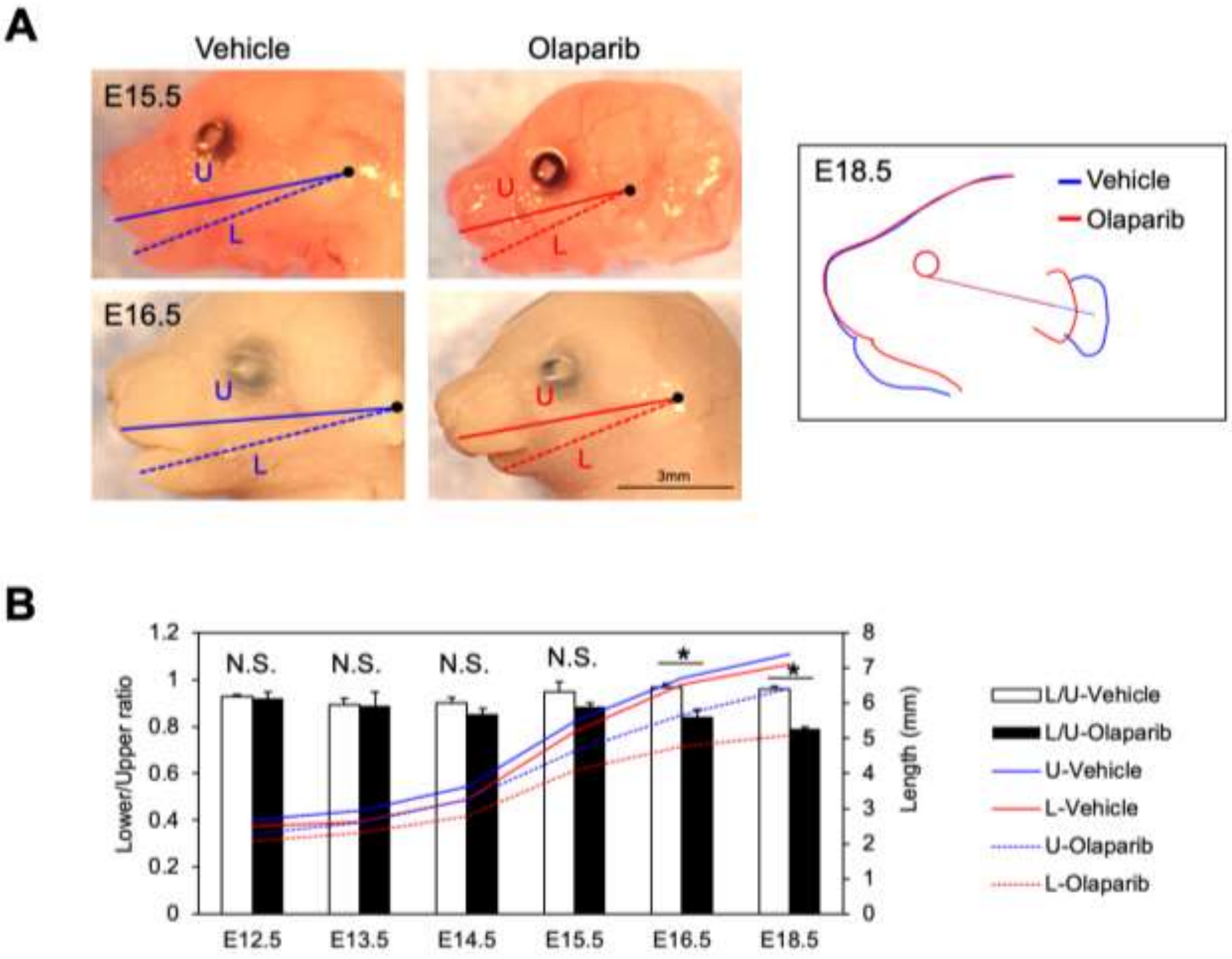

Supplementary Fig. 2. The ratio of maxilla-mandible length of Olaparib-treated mouse embryos.

(A) The landmarks of mandibular length (L) and maxillary length (U). Schematic illustration of a lateral superimposition of Olaparib-treated (red)/Vehicle-treated (blue) embryo at E18.5. **(B)** The analysis of maxilla (U-Vehicle: blue line, U-Olaparib: blue broken line) and mandible length (L-Vehicle: red line, L-Olaparib: red broken line), and maxilla-mandible ratio (white: L/U-Vehicle, black: L/U-Olaparib). Data are represented as mean±SD, n=6 in each group. *$p<0.05$; N.S., not significant.

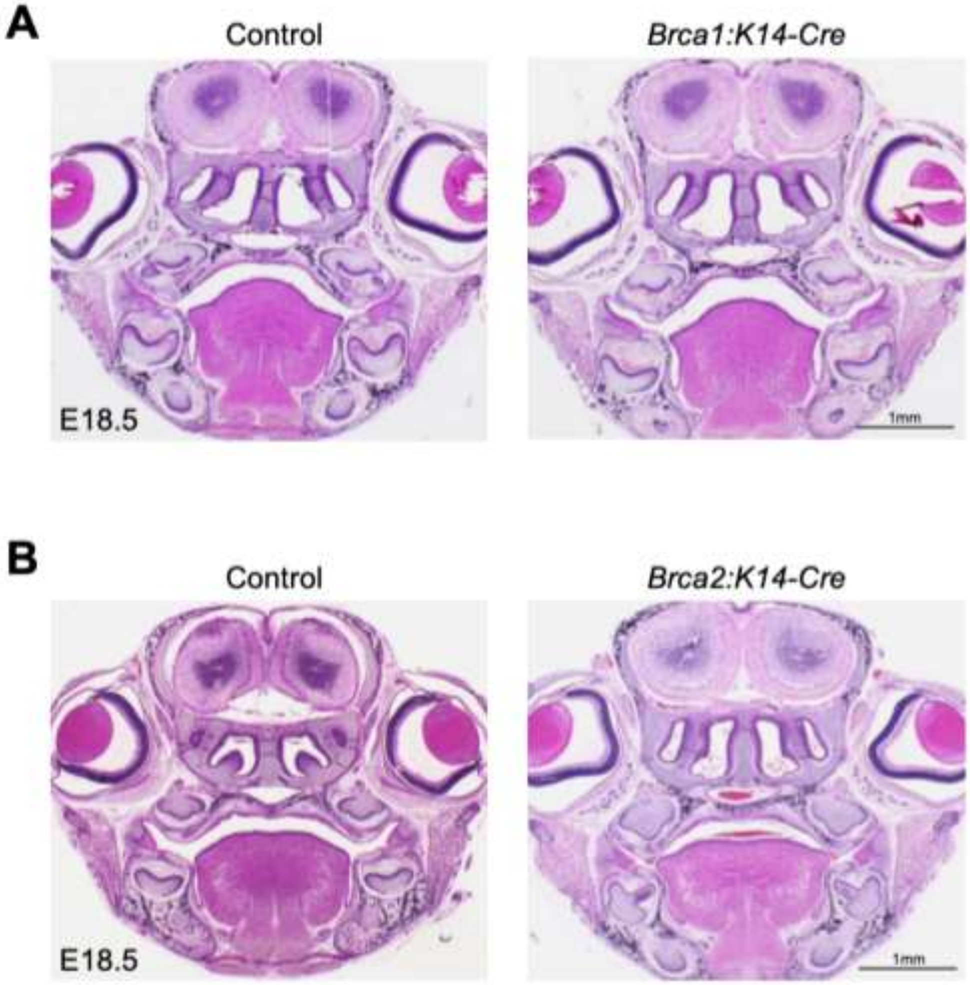

Supplementary Fig. 3. Absence of cleft palate with epithelial cell-specific deletion of *Brca1* and *Brca2* in mice.

(A) H&E staining of coronal section of control and *Brca1:K14-Cre* mice. **(B)** H&E staining of coronal section of control and *Brca2:K14-Cre* mice.

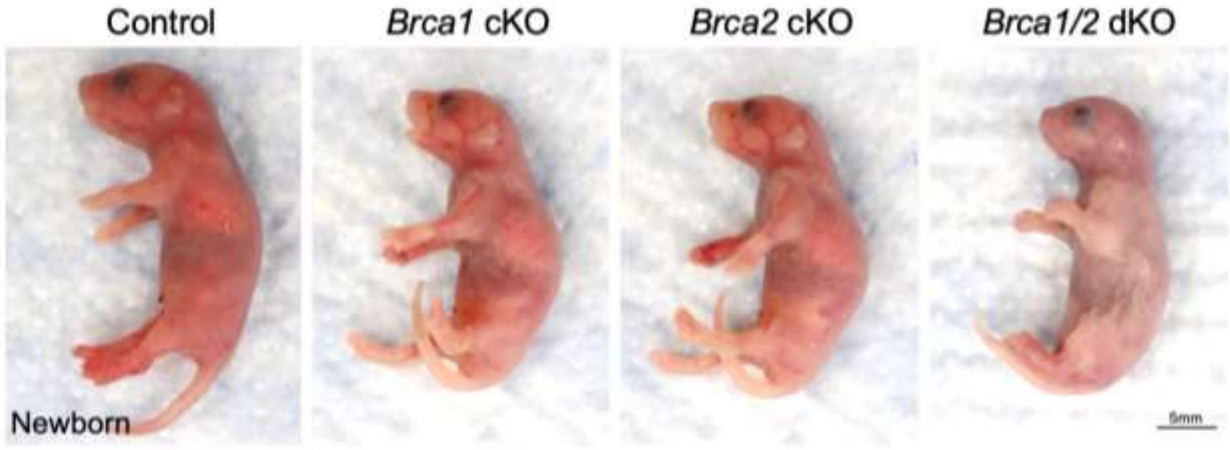

Supplementary Fig. 4. Neonatal death in neural crest-specific *Brca1*, *Brca2* and *Brca1/2* double knockout mice.

Whole-mount view of control, *Brca1:Wnt1-Cre* (*Brca1* cKO), *Brca2:Wnt1-Cre* (*Brca2* cKO), and *Brca1:Brca2:Wnt1-Cre* (*Brca1/2* dKO) newborn mice.

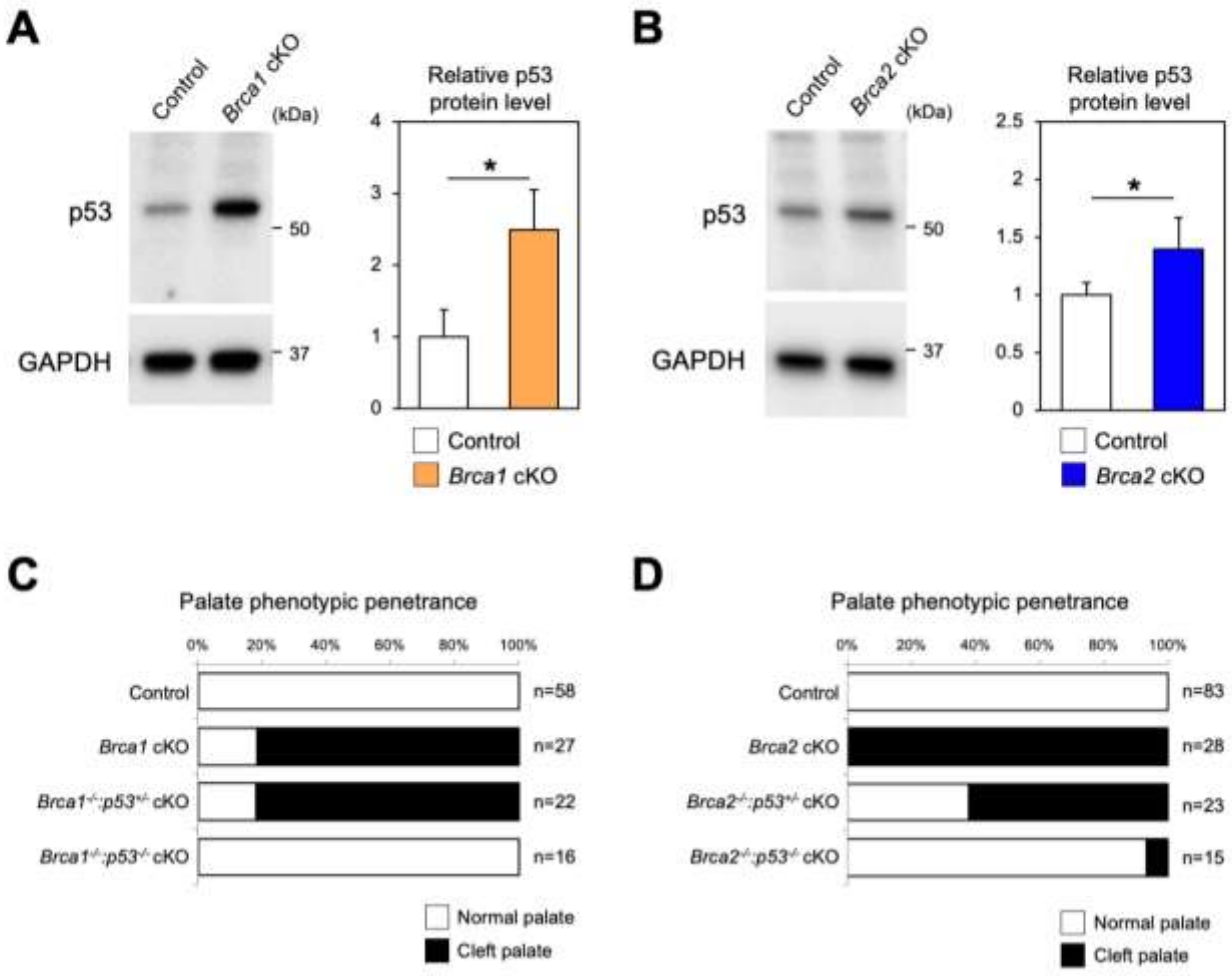

Supplementary Fig. 5. The BRCA1–p53 and BRCA2–p53 pathways play a pivotal role in palatogenesis.

(A, B) Western blotting analysis of palatal tissue from E13.5 embryos. Each sample is from an individual embryo. The chart on the right shows the quantification of the relative p53 protein levels. **(C, D)** Quantification of the phenotypic penetration for each genotype.

Data in (A) and (B) are represented as mean±SD, n=3 in each group. *$p<0.05$.

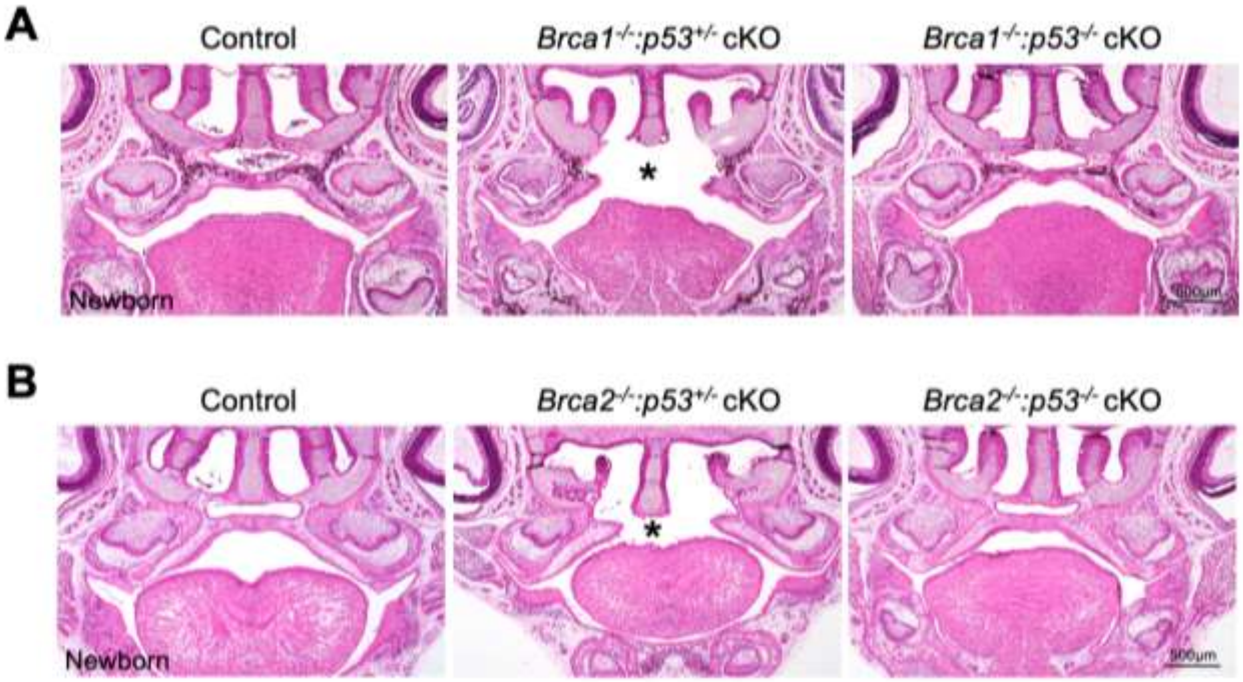

Supplementary Fig. 6. Deletion of p53 rescues cleft palate in *Brca1* and *Brca2* mutant mice.

(A) H&E staining of coronal section of control, *Brca1*$^{-/-}$*:p53*$^{+/-}$ cKO, and *Brca1*$^{-/-}$*:p53*$^{-/-}$ cKO mice. Asterisk shows cleft palate. **(B)** H&E staining of coronal section of control, *Brca2*$^{-/-}$*:p53*$^{+/-}$ cKO, and *Brca2*$^{-/-}$*:p53*$^{-/-}$ cKO mice. Asterisk shows cleft palate.

Intraperitoneal injection (Once/day, daily)			Dead		Alive			
					Normal palate		Cleft palate	
**(A)**	E8.5-15.5	50mg/kg Olaparib	19	(90.5%)	0	(0%)	2	(9.5%)
**(B)**	E10.5-15.5	50mg/kg Olaparib	0	(0%)	3	(9.1%)	30	(90.9%)
**(C)**	E11.5-15.5	50mg/kg Olaparib	0	(0%)	9	(100%)	0	(0%)
**(D)**	E12.5-15.5	50mg/kg Olaparib	0	(0%)	25	(100%)	0	(0%)
**(E)**	E8.5-15.5	5.7% DMSO	0	(0%)	37	(100%)	0	(0%)
**(F)**	E10.5-15.5	5.7% DMSO	0	(0%)	34	(100%)	0	(0%)

Supplementary Table 1. Summary of cleft palate phenotypes following treatment with Olaparib at different timepoints.

(A) 90.5% of Olaparib-treated embryos (50mg/kg, E8.5-15.5, daily) were dead at E16.5.

(B) 90.9% of Olaparib-treated embryos (50mg/kg, E10.5-15.5, daily) were alive and showed cleft palate at E16.5. **(C, D)** All of the Olaparib-treated embryos (50mg/kg, E11.5 or E12.5-15.5, daily) were alive and showed normal palate development at E16.5. **(E, F)** All of the vehicle-treated embryos (5.7%DMSO, E8.5-E15.5 or E10.5-15.5, daily) were alive and showed normal palate development at E16.5.
